# Supplementary material for: Use of soybean as an alternative protein source for welfare-orientated production of American alligators (Alligator mississippiensis)
Source: PeerJ. 2023 Oct 26;11:e16321. doi: 10.7717/peerj.16321 (PMC10613434; doi:10.7717/peerj.16321)
Supplement: Supplemental Information 1 [file peerj-11-16321-s001.docx]

**50% Gator (X-FAT)**

Crude Protein- minimum 50%

Crude Fat- minimum 12%

Crude Fiber- maximum 4%

Phosphorus- minimum 1%

Animal Protein Products

Grain Products

Processed Grain By-Products

Animal Fat (preserved with BHT)

Plant Protein Products

L-Lysine

DL-Methionine

Propionic Acid (a Preservative)

L-escorbyl-2-polyphosphate

L-Threonine

Choline Chloride

Potassium Chloride

Fish oil (Preserved with BHT)

Inositol

Zinc Sulfate

Vitamin E Supplement

Magnesium Oxide

Manganese Sulfate

Niacin Supplement

d-Calcium Pantothenate

Biotin

Menadione Sodium

Bisulfite Complex (source of Vitamin K activity)

Manganese Amino Acid Complex

Zinc Amino Acid

Complex Copper Sulfate

Vitamin A Supplement

Vitamin D3 Supplement

Sodium Selenite

Riboflavin Supplement

Pyrdoxine Hydrochloride

Folic Acid

Vitamin B12 Supplement

Thiamine

Mononitrate

Ethylenediamine Dihydroiodide,

Cobalt Sulfate

Citric Acid (as a preservative)

Butylated Hydroxyanisole (BHA) (as a preservative)

Ethoxyquin (as a preservative).

**50% Gator (X-FAT) Soy Test**

Crude Protein- minimum 50%

Crude Fat- minimum 12%

Crude Fiber- maximum 4%

Phosphorus- minimum 1%

Plant Protein Products

Grain Products

Processed Grain By-Products

Animal Fat (preserved with BHT)

L-Lysine

DL-Methionine

Propionic Acid (a Preservative)

L-escorbyl-2-polyphosphate

L-Threonine

Choline Chloride

Potassium Chloride

Fish oil (Preserved with BHT)

Inositol

Zinc Sulfate

Vitamin E Supplement

Magnesium Oxide

Manganese Sulfate

Niacin Supplement

d-Calcium Pantothenate

Biotin

Menadione Sodium

Bisulfite Complex (source of Vitamin K activity)

Manganese Amino Acid Complex

Zinc Amino Acid

Complex Copper Sulfate

Vitamin A Supplement

Vitamin D3 Supplement

Sodium Selenite

Riboflavin Supplement

Pyrdoxine Hydrochloride

Folic Acid

Vitamin B12 Supplement

Thiamine

Mononitrate

Ethylenediamine Dihydroiodide,

Cobalt Sulfate

Citric Acid (as a preservative)

Butylated Hydroxyanisole (BHA) (as a preservative)

Ethoxyquin (as a preservative)
